# The association between omega-3 supplementation and cognitive decline in older adults

**DOI:** 10.1016/j.tjpad.2026.100569

**Published:** 2026-04-17

**Authors:** Zheng-Bin Liao, Zi-Cheng Hu, Gui-Hua Zeng, Jia Chen, Xin-Peng Li, Yu-Hui Liu, Xiu-Qing Yao, Ye-Ran Wang

**Affiliations:** a Department of Clinical Medicine, School of Basic Medicine, Third Military Medical University, Chongqing, 400038, China; b Department of Neurology, The First Affiliated Hospital of Chongqing Medical University, Chongqing, 400016, China; c Department of Neurology and Centre for Clinical Neuroscience, Daping Hospital, Third Military Medical University, Chongqing, 400042, China; d Department of Rehabilitation, The Second Affiliated Hospital of Chongqing Medical University, No. 74 Linjiang Road, Yuzhong District, Chongqing, 400010, China

**Keywords:** Alzheimer, Omega-3, Cognitive, Amyloid-β, Tau, synaptic dysfunction

## Abstract

**Background:**

While omega-3 fatty acid supplementation is widely used for cognitive protection, its efficacy remains controversial, and its impact on core Alzheimer's disease (AD) pathologies in humans is not well-established.

**Methods:**

This longitudinal study utilized data from the Alzheimer's Disease Neuroimaging Initiative (ADNI). We employed linear mixed-effects models to assess the association between omega-3 supplementation and longitudinal cognitive decline, and mediation analyses to examine whether this relationship was mediated by core AD pathologies (Aβ-PET, tau-PET, T1-MRI, FDG-PET).

**Results:**

Omega-3 supplementation was associated with significantly accelerated cognitive decline, as evidenced by a faster decrease in MMSE scores (β = -0.266, *p* < 0.001) and a faster increase in both ADAS-Cog13 (β = 0.823, *p* < 0.001) and CDR-SB scores (β = 0.205, *p* < 0.001). This association was not mediated by Aβ deposition, tau pathology, or gray matter atrophy. Instead, longitudinal FDG hypometabolism within AD-vulnerable regions served as a significant mediating pathway, accounting for 30.8%, 40.8%, and 19.0% of the total effect on the decline in MMSE, ADAS-Cog13, and CDR-SB, respectively.

**Conclusions:**

Omega-3 supplementation may be associated with accelerated cognitive decline in older adults, potentially through adverse effects on cerebral synaptic function rather than classical AD proteinopathies. These findings challenge the prevailing view of omega-3 as uniformly beneficial and highlight the need for a cautious reassessment of its widespread use for cognitive protection.

## 1 Background

Alzheimer’s disease (AD), the leading cause of dementia, imposes a growing global public health burden [1]. Individuals with AD initially present with short-term memory difficulties and amnestic cognitive impairment. As the disease progresses, they gradually develop deficits across multiple cognitive domains, including visuospatial processing, attention, language, and executive function, ultimately leading to significant loss of daily living abilities [2,3]. The core pathological hallmarks of AD include the extracellular deposition of β-amyloid (Aβ) plaques, the intracellular aggregation of hyperphosphorylated tau protein, and subsequent neurodegeneration [4]. Research indicates that Aβ deposition begins more than 20 years before the onset of clinical symptoms [5], and is regarded as the initiating event in the AD pathological cascade [6,7]. In contrast, the propagation of pathological tau is more closely paralleled to cognitive decline and is considered a key driver of neuronal dysfunction [8,9]. These pathological proteins can be detected *in vivo* using molecular imaging techniques, namely amyloid β positron emission tomography (Aβ PET) [10] and tau PET [11]. Neurodegeneration, the irreversible loss of neurons and synapses, is the direct pathological basis for cognitive deficits [12]. This can be captured by gray matter volume (GMV) atrophy on structural magnetic resonance imaging (sMRI) [13] and synaptic dysfunction on fluorodeoxyglucose–PET (FDG PET) [14].

However, despite substantial advances in understanding the mechanisms of AD, effective therapeutic strategies directly targeting its core pathology remain limited. Consequently, identifying modifiable risk factors to prevent or delay cognitive decline is of critical public health importance [15,16]. Recently, dietary interventions [17], particularly the role of omega-3 polyunsaturated fatty acids (PUFAs) in slowing cognitive decline, have garnered widespread attention. Omega-3 fatty acids are a class of PUFAs characterized by a double bond at the omega-3 position, primarily including docosahexaenoic acid (DHA), eicosapentaenoic acid (EPA), and alpha-linolenic acid (ALA) [18]. Numerous animal studies and observational studies have suggested that omega-3 fatty acids possess neuroprotective properties and may slow cognitive decline [[19], [20], [21], [22]]. Nevertheless, this benefit has not been consistently confirmed in randomized controlled trials (RCTs) [23]. Most RCTs report no cognitive benefit from omega-3 fatty acids supplementation in AD patients [[24], [25], [26], [27]], and one recent trial even suggested adverse effects on synaptic integrity [28].

This ongoing controversy underscores the need to clarify whether omega-3 supplementation influences cognitive trajectories and its underlying biological pathways [23]. To address this issue, this study utilizes data from the Alzheimer's Disease Neuroimaging Initiative (ADNI) database to investigate the association between omega-3 supplementation and longitudinal cognitive decline and to test whether this relationship is mediated by core AD pathologies, including Aβ, tau, and neurodegeneration. By elucidating these specific neurobiological pathways, we aim to provide clinical evidence that informs the debate regarding the role of omega-3 fatty acids supplementation in slowing cognitive decline.

## 2 Methods

### 2.1 Participants

Data used in the preparation of this article were obtained from the Alzheimer’s Disease Neuroimaging Initiative (ADNI) database (adni.loni.usc.edu). The ADNI was launched in 2003 as a public-private partnership, led by Principal Investigator Michael W. Weiner, MD. The primary goal of ADNI has been to test whether serial magnetic resonance imaging (MRI), positron emission tomography (PET), other biological markers, and clinical and neuropsychological assessment can be combined to measure the progression of mild cognitive impairment (MCI) and early AD. The study included participants aged 55 to 90 years, encompassing cognitively normal (CN) older adults, individuals with significant memory concern (SMC), and patients diagnosed with MCI or dementia due to AD. Comprehensive assessments were performed, encompassing clinical evaluations, neuropsychological testing, genetic analyses, MRI and PET imaging, as well as detailed medical and personal history interviews. All participants provided written informed consent. The study protocol was approved by the Institutional Review Board of Daping Hospital, Third Military Medical University.

### 2.2 Cognitive measurements

This study employed three commonly used cognitive scales to assess longitudinal trajectories associated with omega-3 supplementation: the Mini-Mental State Examination (MMSE), the Alzheimer’s Disease Assessment Scale–Cognitive Subscale 13 (ADAS-Cog13), and the Clinical Dementia Rating–Sum of Boxes (CDR-SB). The MMSE (range 0–30) is a brief global screening tool for cognitive impairment, with higher scores indicating better cognitive performance [29]. The ADAS-Cog13 (range 0–85) is widely used to evaluate therapeutic efficacy and disease progression, covering multiple domains including memory, language, praxis, attention, and other cognitive functions, with higher scores reflecting greater impairment [30]. The CDR-SB (range 0–18), a quantitative extension of Clinical Dementia Rating (CDR), like the ADAS-Cog13, higher scores indicate worse performance [31].

### 2.3 Information about omega-3 supplementation and *APOE* ε4 carrier status

The apolipoprotein E (*APOE*) genotype was determined based on two single-nucleotide polymorphisms (SNPs), rs429358 and rs7412, which define the ε2, ε3, and ε4 alleles. Participants carrying at least one ε4 allele were classified as *APOE* ε4 carriers, while all others were classified as non-carriers. Omega-3 supplementation encompassed several types, such as fish oil, flaxseed oil, and krill oil. For analysis, the exposure time point for omega-3 users was defined as the first follow-up visit after a participant reported initiating supplementation. Visits before and after this time point were classified as the non-exposure and exposure periods, respectively.

### 2.4 MRI and PET imaging processing

Detailed acquisition parameters for 3D T1-weighted MRI and PET can be accessed from the ADNI information portal (http://adni-info.org). Structural MRI data were processed using FreeSurfer (version 4.4.0) for cortical and subcortical segmentation. From the complete set of segmentations generated by FreeSurfer, regional GMV was specifically extracted for 68 cortical regions of interest (ROIs) defined by the Desikan-Killiany atlas for use in our regional analyses [32]. Because gray matter volume (GMV), Aβ, tau, and fluorodeoxyglucose (FDG) abnormalities manifest in distinct brain regions, composite ROIs were defined for each pathology. For gray matter volume, the meta-region of interest gray matter volume (Meta-ROI GMV) was defined as a composite of AD-specific atrophy regions, including the entorhinal cortex, fusiform gyrus, inferior temporal gyrus, and middle temporal gyrus [33]. The composite index was calculated by summing GMV across these regions. All PET images, including Aβ PET (¹⁸F-florbetapir, FBP), tau PET (¹⁸F-flortaucipir, FTP), and FDG PET, were coregistered to the closest structural MRI. Regional standardized uptake value ratio (SUVR) values for FBP and FTP across the 68 Desikan-Killiany ROIs were extracted in the native T1 space for regional analyses. Composite SUVR measures were also calculated for each tracer. The Meta-ROI FBP SUVR was calculated as the volume-weighted average uptake in the frontal, cingulate, lateral parietal, and lateral temporal cortices [34], normalized to whole cerebellum uptake. The Meta-ROI FTP SUVR was calculated as the volume-weighted average uptake in the entorhinal cortex, amygdala, fusiform gyrus, inferior temporal gyrus, and middle temporal gyrus [33], normalized to the inferior cerebellar gray matter. The Meta-ROI FDG SUVR was calculated as the mean uptake in the left angular gyrus, right angular gyrus, bilateral posterior cingulate cortex, left inferior temporal gyrus, and right inferior temporal gyrus [35], normalized to the pons and cerebellar vermis.

### 2.5 Statistical analysis

All statistical analyses were performed in R (version 4.5.0). To control for baseline confounding, propensity score matching (PSM) was conducted using 1:2 nearest-neighbor matching with a caliper of 0.2. The propensity score model incorporated age, sex, *APOE* ε4 status, and diagnosis as covariates. Continuous variables were summarized as medians with interquartile ranges (IQR), and categorical variables as counts with percentages. Group differences at baseline were assessed using two-sided Mann–Whitney U tests or Fisher’s exact tests. To further assess covariate balance after propensity score matching, standardized mean differences (SMDs) were calculated, with an SMD < 0.1 indicating adequate balance.

To assess the effects of omega-3 supplementation on cognitive trajectories and core AD pathologies, linear mixed effects models (LMMs) were fitted to estimate whether longitudinal changes (slopes, Δ) in cognitive scores and imaging biomarkers differed between the omega-3 exposed and non-exposed groups. The outcomes included cognitive scores (MMSE, ADAS-Cog13, CDR-SB), composite biomarker measures (Meta-ROI FBP SUVR, Meta-ROI FTP SUVR, Meta-ROI GMV, Meta-ROI FDG SUVR), and the regional biomarker measures (including FBP SUVR, FTP SUVR, and GMV) across 68 ROIs. Models were adjusted for age, sex, *APOE* ε4 status, and diagnosis, with random intercept and slope for each participant (except for the FTP SUVR model, which included a random intercept only). Notably, the model with GMV as the dependent variable was additionally adjusted for intracranial volume (ICV) [36]. Continuous variables with large scale differences (GMV, ICV, and Age) were standardized (mean = 0, SD = 1) prior to modeling. The model is as follows:

model:CognitivescoresORImagingbiomarkers∼Time*Omega3status+Age+Sex+APOEε4status+Diagnosis+(1+Time|Individual)

For the analysis assessing the association between omega-3 supplementation and the 68 regional biomarkers, we corrected for multiple comparisons by controlling the false discovery rate (FDR) using the Benjamini-Hochberg procedure; an adjusted p-value < 0.05 was considered statistically significant.

To assess the possibility of reverse causality, where the decision to initiate omega-3 supplementation might be influenced by an individual’s pre-existing health trajectory, We compared the longitudinal trajectories during the pre-supplementation period in future omega-3 users with those in matched non-users. This comparison included the cognitive scores and the key neuroimaging biomarkers.

We then conducted mediation analyses to provide an integrative evaluation of the associations among omega-3 supplementation, core AD pathologies, and cognitive decline, and to further quantify the specific mediating contribution of core AD pathologies to the relationship between omega-3 supplementation and cognitive decline. Indirect effects were estimated using the product-of-coefficients method (β_1_ × β_2_) within a longitudinal mediation framework, with 5,000 bootstrap iterations employed to derive 95% confidence intervals (CIs) for both indirect and total effects. Indirect and total effects were considered statistically significant if the 95% CI did not include zero. All mediation models were adjusted for age, sex, *APOE* ε4 status, and diagnosis.

## 3 Results

### 3.1 Demographics

**Fig. 1:**
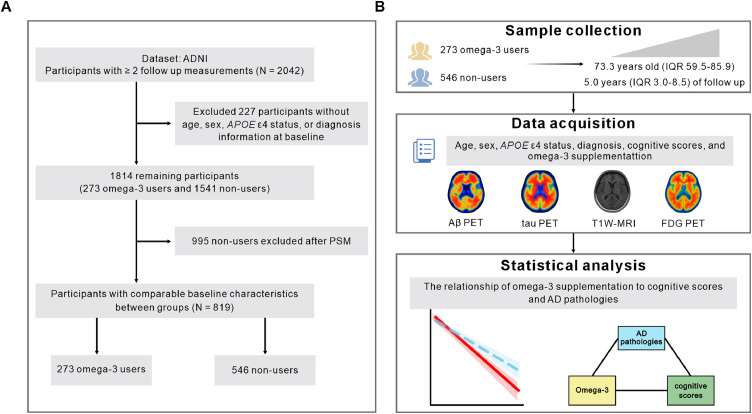
Participant selection flowchart and study design. Flowchart of the study participants derived from the ADNI (A). Conceptual framework outlining the study pipeline (B). ADNI, Alzheimer's Disease Neuroimaging Initiative; PSM, Propensity Score Matching; IQR, interquartile range; AD, Alzheimer’s disease; T1W-MRI, T1-weighted magnetic resonance imaging; Aβ, amyloid-β; FDG, Fluorodeoxyglucose; PET, positron emission tomography.

The participant selection procedure is illustrated in Fig. 1. A total of 1,814 participants were initially identified from the ADNI cohort, comprising 1,541 omega-3 non-users and 273 users (Table S1). After PSM for age, sex, *APOE* ε4 status, and diagnosis, the final cohort included 546 matched non-users and 273 users, with a median follow up duration of 5 years (IQR 3-8.5). With respect to omega-3 supplementation, the primary formulation reported was fish oil (Fig. S1).

The baseline characteristics of the matched sample are summarized in Table 1. Missing data for the variables are presented in the 'Missing' row below. Specifically, omega-3 users and non-users had comparable median age, sex composition, frequencies of *APOE* ε4 carriers, diagnosis composition, median education years, median ADAS-Cog13 scores, median FDG-SUVR, proportions of subjects with stroke, insomnia, depression, anxiety, and Parkinson’s disease.

**Table 1:** Demographic characteristics of participants.

	Group			
	Omega-3 non-users	Omega-3 Users		
Characteristic	N = 546	N = 273	SMD	P.value
Age,median(IQR,range)	73.5 (68.2, 78.5)	73.0 (69.3, 77.4)	0.05	0.543
Gender,n(%)			0.05	0.544
Male	337 (61.7%)	162 (59.3%)		
Female	209 (38.3%)	111 (40.7%)		
*APOE* ε4,n(%)			0.04	0.655
No	298 (54.6%)	154 (56.4%)		
Yes	248 (45.4%)	119 (43.6%)		
Diagnosis,n(%)			0.07	0.717
CN	158 (28.9%)	79 (28.9%)		
SMC	10 (1.8%)	3 (1.1%)		
EMCI	134 (24.5%)	65 (23.8%)		
MCI	187 (34.2%)	95 (34.8%)		
LMCI	27 (4.9%)	14 (5.1%)		
AD	30 (5.5%)	17 (6.2%)		
Education,median(IQR,range)	16.0 (14.0, 18.0)	16.0 (14.0, 18.0)	-0.03	0.761
Missing	371	104		
ADAS-Cog13,median(IQR,range)	12.0 (8.0, 16.0)	12.0 (8.0, 17.0)	0.01	0.932
Missing	227	167		
FDG SUVR,median(IQR,range)	1.2 (1.1, 1.3)	1.2 (1.1, 1.3)	-0.03	0.913
Missing	148	82		
Stroke,n(%)			0.12	>0.999
No	269 (99.3%)	56 (100.0%)		
Yes	2 (0.7%)	0 (0.0%)		
Missing	275	217		
Parkinson,n(%)			0.14	0.251
No	320 (100.0%)	106 (99.1%)		
Yes	0 (0.0%)	1 (0.9%)		
Missing	226	166		
Insomnia,n(%)			0.08	0.804
No	40 (81.6%)	40 (78.4%)		
Yes	9 (18.4%)	11 (21.6%)		
Missing	497	222		
Anxiety,n(%)			0.15	0.363
No	241 (88.9%)	47 (83.9%)		
Yes	30 (11.1%)	9 (16.1%)		
Missing	275	217		
Depression,n(%)			0.01	>0.999
No	251 (92.6%)	52 (92.9%)		
Yes	20 (7.4%)	4 (7.1%)		
Missing	275	217		

Note. Data are presented as median (interquartile range) for continuous variables and counts (%) for categorical variables. Group comparisons were conducted using the Mann–Whitney U test or Fisher’s exact test, as appropriate. Standardized mean differences (SMD) were also calculated to assess covariate balance after matching, with SMD < 0.1 indicating adequate balance.

Abbreviations: IQR, interquartile range; *APOE*, apolipoprotein E; CN, cognitively normal; EMCI, early mild cognitive impairment; LMCI, late mild cognitive impairment; SMC, significant memory concern; AD, Alzheimer’s disease; ADAS-Cog13, Alzheimer’s Disease Assessment Scale-Cognitive subscale 13; FDG, fluorodeoxyglucose; SUVR, standardized uptake value ratio.

### 3.2 Association between omega-3 supplementation and cognitive trajectories

**Fig. 2:**
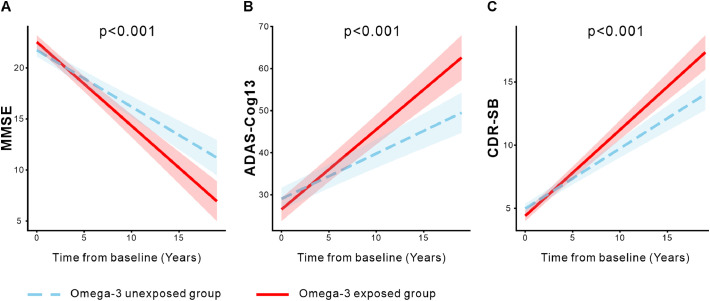
Longitudinal cognitive trajectories associated with omega-3 supplementation. Comparisons of longitudinal changes in MMSE (A), ADAS-Cog13 (B), and CDR-SB (C) scores between omega-3 exposed (red) and unexposed (blue) groups. Linear mixed-effects model fits are shown with 95% confidence intervals. All analyses were adjusted for age, sex, *APOE* ε4 status, and diagnosis. MMSE, Mini-Mental State Examination; ADAS-Cog13, Alzheimer’s Disease Assessment Scale-Cognitive Subscale 13; CDR-SB, Clinical Dementia Rating-Sum of Boxes.

Linear mixed-effects models, adjusted for age, sex, *APOE* ε4 status, and diagnosis, revealed a significant time-by-omega-3 interaction (Fig. 2). The β coefficients for the interaction term represent the additional annual rate of change associated with omega-3 exposure, over and above the rate observed in the unexposed group. Specifically, individuals with omega-3 supplementation exhibited a faster decline in MMSE scores (β = −0.266, 95% CI [−0.360, −0.173], p < 0.001; Fig. 2A), a faster increase in ADAS-Cog13 scores (β = 0.823, 95% CI [0.562, 1.080], p < 0.001; Fig. 2B), and a faster increase in CDR-SB scores (β = 0.205, 95% CI [0.146, 0.264], p < 0.001; Fig. 2C), indicating more rapid cognitive deterioration over time among omega-3 exposed group.

### 3.3 Association between omega-3 supplementation and neuroimaging biomarkers

**Fig. 3:**
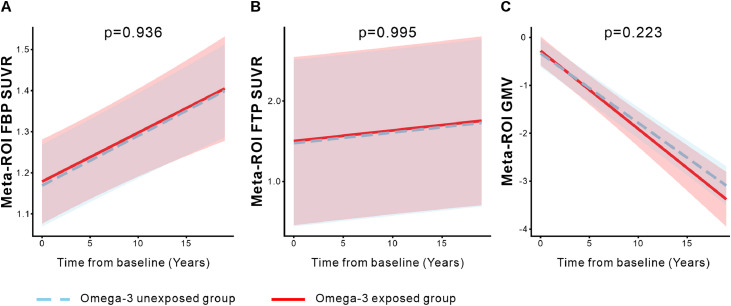
Longitudinal changes in Aβ deposition (Meta-ROI FBP SUVR), tau aggregation (Meta-ROI FTP SUVR), and gray matter volume (Meta-ROI GMV) were not associated with omega-3 supplementation. Trajectories for the omega-3 exposed (red) and unexposed (blue) groups are shown for Aβ (A), tau (B), and GMV (C), with lines representing linear mixed effects model fits and shaded areas indicating 95% confidence intervals. All analyses were adjusted for age, sex, APOE ε4 status, and diagnosis. Aβ, amyloid-β; FBP, ¹⁸F-florbetapir; FTP, ¹⁸F-flortaucipir; Meta-ROI, meta-region of interest; GMV, gray matter volume; SUVR, standardized uptake value ratio.

**Fig. 4:**
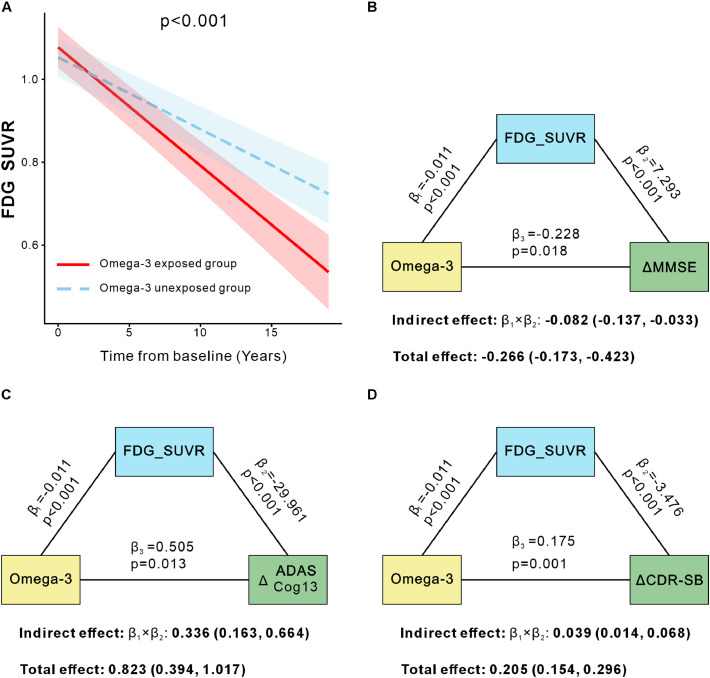
FDG hypometabolism mediates the association between omega-3 supplementation and cognitive decline. Comparison of longitudinal change in the composite regions FDG SUVR between omega-3 exposed(red) and omega-3 unexposed(blue) groups (A). The mediation effect of FDG SUVR in composite regions on the association between omega-3 and ΔMMSE, ΔADAS-Cog13 and ΔCDR-SB (B, C, D). Path coefficients (β) from the mediation models are shown. The indirect effect is represented by β1 × β2, and the direct effect by β3. Total effects were calculated from linear mixed model of cognitive scores. 95% confidence intervals for the indirect and direct effects were estimated using a bootstrap procedure with 5000 iterations. MMSE, Mini-Mental State Examination; ADAS-Cog13, Alzheimer’s Disease Assessment Scale-Cognitive subscale 13; CDR-SB, Clinical Dementia Rating Sum of Boxes; SUVR, standardized uptake value ratio.

Compared with non-exposed group, omega-3 supplementation was not associated with longitudinal changes in Aβ deposition (Meta-ROI FBP SUVR: β = 0.000, 95% CI [−0.006, 0.005]; Fig. 3A); tau aggregation (Meta-ROI FTP SUVR: β = 0.000, 95% CI [−0.015, 0.015]; Fig. 3B), or GMV atrophy (Meta-ROI GMV: β = −0.018, 95% CI [−0.046, 0.011]; Fig. 3C). Furthermore, analyses across all 68 individual ROIs revealed no significant associations with Aβ, tau, or GMV after FDR correction (all FDR-adjusted p-values > 0.05; Table S2-4). In contrast, omega-3 exposed group exhibited a significantly greater longitudinal decline in composite regional glucose metabolism, as indexed by Meta-ROI FDG SUVR (β = −0.011, 95% CI [−0.016, −0.006]; Fig. 4A), suggesting a potential link to synaptic injury.

### 3.4 No pre-existing differences in cognitive or FDG-SUVR trajectories before the initiation of omega-3 supplementation

To assess the potential for reverse causality, we compared longitudinal trajectories during the pre-supplementation period in future omega-3 users with those of matched non-users across cognitive (MMSE, ADAS-Cog13, CDR-SB) and metabolic (Meta-ROI FDG SUVR) measures. No significant differences were observed, indicating no evidence of pre-existing decline in future users relative to non-users (MMSE: β = –0.002, 95% CI [–0.223, 0.221]; ADAS-Cog13: β = –0.390, 95% CI [–1.061, 0.273]; CDR-SB: β = –0.139, 95% CI [–0.305, 0.025]; Meta-ROI FDG SUVR: β = 0.006, 95% CI [–0.002, 0.013]) (Fig. S2).

### 3.5 FDG hypometabolism mediated the association between omega-3 supplementation and accelerated cognitive decline

We next conducted mediation analyses to test whether hypometabolism in the composite regions mediated the association between omega-3 supplementation and cognitive decline. Results revealed a significant indirect effect through FDG hypometabolism for all three cognitive measures, indicating that diminished cerebral glucose metabolism partially explained the accelerated cognitive deterioration observed in omega-3 exposed group (Fig. 4). Specifically, the indirect pathway via FDG hypometabolism accounted for 30.8% of the total effect of omega-3 supplementation on the decline in MMSE scores (β_indirect = −0.082, 95% CI [−0.137, −0.033]; Fig. 4B), 40.8% for the increase in ADAS-Cog13 scores (β_indirect = 0.336, 95% CI [0.163, 0.664]; Fig. 4C), and 19.0% for the increase in CDR-SB scores (β_indirect = 0.039, 95% CI [0.014, 0.068]; Fig. 4D). These findings support the notion that synaptic dysfunction, as reflected by reduced cerebral glucose metabolism, serves as a key pathway linking omega-3 supplementation to more rapid cognitive decline.

## 4 Discussion

In this longitudinal study leveraging data from the ADNI cohort, we examined the association between omega-3 supplementation and cognitive decline, and further explored potential mediating pathways using multimodal imaging biomarkers. Contrary to the prevailing hypothesis of a neuroprotective role, omega-3 supplementation was associated with accelerated cognitive decline, suggesting that the cognitive impact of omega-3 in aging brain may be more complex and context dependent than previously assumed. Importantly, this association was not mediated by the canonical AD pathways of Aβ deposition, tau aggregation, or gray matter atrophy. Instead, longitudinal reductions in FDG SUVR within AD-specific hypometabolic regions significantly mediated the relationship between omega-3 exposure and cognitive deterioration, suggesting that synaptic dysfunction may underlie this paradoxical effect.

To contextualize the clinical magnitude of the observed effect, we compared the additional annual change in cognitive scores associated with omega-3 supplementation with the typical annual progression rates reported in AD patients (MMSE: -3.4 points/year; ADAS-Cog: +5.5 points/year; CDR-SB: +1.91 points/year) [[37], [38], [39]]. The accelerated decline linked to omega-3 use corresponded to approximately 7.8%, 15.0%, and 10.7% of the annual AD progression for MMSE, ADAS-Cog, and CDR-SB, respectively. Notably, the accelerated decline observed in omega-3 users does not appear to be driven by reverse causality. The cognitive trajectories and cerebral glucose metabolism during the pre-supplementation period in future omega-3 users were not different from, and were even directionally slightly better than, those of matched non-users. This finding indicates that the detrimental association we observed emerged after the reported initiation of supplementation, suggesting that factors specifically associated with omega-3 exposure may have contributed to the long-term acceleration of decline. However, this analysis may miss subtle trajectories that prompt individuals to start supplements.

The relationship between omega-3 supplementation and cognitive outcomes has been extensively investigated, yet evidence remains inconsistent. Proposed neuroprotective mechanisms, such as modulation of neurotransmitter systems, anti-inflammatory effects, regulation of membrane fluidity, and support for neurodevelopment, are largely derived from animal studies [[40], [41], [42]]. Although human observational data often align with these beneficial effects [[20], [21], [22]], RCTs, which offer stronger causal inference, have generally failed to demonstrate cognitive benefits [[24], [25], [26], [27],^43^]. This unresolved conflict underscores a critical gap in our understanding upon this issue. The biological pathways through which omega-3 influences cognitive trajectories in humans remain incompletely elucidated. It further raises the possibility that omega-3 supplementation may exert adverse effects that offset its potential benefits, resulting in an overall neutral impact on cognition. Notably, a small RCT reported a counterintuitive increase in neurofilament light chain (NfL), a biomarker of axonal and synaptic injury, among omega-3 exposed group compared with the placebo group, although no significant cognitive decline was observed [28]. The authors did not fully explain this paradoxical finding. Our findings extend this line of evidence by demonstrating that omega-3 supplementation was associated with longitudinal FDG hypometabolism in AD-vulnerable regions, suggestive of persistent synaptic dysfunction, yet without influencing the trajectories of Aβ deposition, tau pathology, or gray matter atrophy—consistent with previous studies [44].

The persistent FDG hypometabolism observed in our study may be mechanistically linked to oxidative stress and mitochondrial dysfunction. Although omega-3 polyunsaturated fatty acids possess well-documented anti-inflammatory and antioxidant properties, their highly unsaturated structure—particularly that of DHA—renders them the most peroxidation-susceptible lipids in brain mitochondria [45]. The resulting peroxidation products can directly compromise mitochondrial membrane integrity, inhibit oxidative phosphorylation, impair energy metabolism, and paradoxically exacerbate reactive oxygen species production, thereby establishing a self-reinforcing vicious cycle [46]. This dual nature of omega-3 fatty acids has been well-documented in aging rodent models, where supplementation attenuated inflammatory pathways while simultaneously enhancing oxidative stress markers and senescence-associated protein expression [47].

The net effects of omega-3 supplementation on brain health are likely governed by a delicate balance between its neuroprotective benefits and potential deleterious effects, modulated by dosage and individual baseline characteristics (e.g., plasma/brain omega-3 levels, preexisting cerebral oxidative burden, and underlying neuropathology). A recent systematic review has indicated that low-dose supplementation is associated with net cognitive benefit, whereas high doses may reverse this advantage [48]. Furthermore, individuals with low baseline omega-3 levels tend to derive greater benefit from supplementation [49]. Individuals with elevated cerebral oxidative stress and inflammation often show limited benifits [50]. A systemic analysis which included 24 studies have demonstrated that omega-3 supplementation is ineffective in AD patients [50]. In addition, omega-3 exacerbates α-synuclein accumulation in Parkinson’s disease models [51], and aggravates neuronal damage following cerebral ischemia [52].

Our study differs from previous RCTs that reported neutral results in several key aspects of experimental design. First, regarding the type of supplement, previous RCTs predominantly used EPA and DHA [24,26], whereas our study mostly employed commercially available fish oil, which is associated with a significantly higher risk of oxidation [53]. This may explain why prior RCTs tended to yield neutral findings, while our study observed harmful effects. Second, compared with RCTs whose follow-up duration did not exceed 18 months, our study has a notably longer follow-up (the median follow-up in our cohort was 5 years). This extended observation window allows us to investigate potential sustained or delayed effects of omega-3 on cognitive function, thereby providing a unique lens on its long-term impact.

These findings raise important questions about the traditional assumption that omega-3 fatty acids exert uniformly neuroprotective effects. While prior research has predominantly emphasized their anti-inflammatory and neurotrophic mechanisms, our results suggest a previously underrecognized possibility that omega-3 supplementation may, in some contexts, adversely affect synaptic integrity, ultimately counteracting its short-term benefits. This insight calls for a more nuanced understanding of the role of omega-3 in the aging human brain—beyond a simplistic protective-versus-ineffective framework. From a clinical and public health standpoint, our findings suggest that a more nuanced assessment and further research are warranted, regarding the widespread use of omega-3 supplements for cognitive protection.

This study reported a potentially deleterious association between omega-3 supplementation and cognitive outcomes in older adults. Nevertheless, these findings should be interpreted with caution. First, the observed harmful association presents a paradox, as the typical "healthy user bias" inherent in observational studies would generally favor the detection of a protective effect. One possibility is that this association reflects a genuine adverse effect of omega-3 supplementation as measured in our study. Alternatively, despite our efforts to balance baseline characteristics, including cognitive performance, neuroimaging biomarkers, major comorbidities, and educational level, through propensity score matching, it remains possible that unmeasured factors (e.g., cardiovascular diseases, family history of dementia, chronic pain) may have influenced both omega-3 use and the trajectories of cognitive decline and FDG reduction, thereby leading to an erroneous link between omega-3 supplementation and cognition. Second, due to the retrospective observational design of our study, we were unable to ascertain detailed, longitudinal adherence data (e.g., precise dosage, periods of discontinuation) for the entire follow-up period. This would introduce unavoidable exposure misclassification. Nevertheless, our exposure definition was validated by significantly higher serum omega-3 levels in the exposed group compared with the unexposed group (Fig. S3), suggesting that the definition was generally reasonable. Third, compared with Aβ, GMV, FDG, the tau PET dataset had more limited longitudinal data, which may have reduced the statistical power to detect the associations between omega-3 supplementation and tau pathology. In addition, although the sample size was sufficient to detect the primary associations, it may have limited power for exploring finer-grained subgroup heterogeneity or modest interaction effects. Consequently, we were unable to stratify by specific supplement types (e.g., fish oil versus other types). Given that fish oil, the primary supplement type, is particularly susceptible to oxidation [53], the observed harmful association may not generalize to other omega-3 formulations with greater oxidative stability. Finally, the findings of this study may be limited to the present cohort, which consists predominantly of White and highly educated individuals. Whether these findings generalize to other populations remains unclear. Future studies are warranted to elucidate the dose-dependent, context-dependent, and time-dependent dynamics underlying the potential benefits and harms of omega-3 supplementation. Key contextual factors to prioritize include baseline diagnosis, APOE ε4 status, and baseline omega-3 level.

## Ethics approval and consent to participate

All participants provided written informed consent in the ADNI cohort. The study was conducted in accordance with the Declaration of Helsinki, and the protocol was approved by the Institutional Review Board of Daping Hospital.

## Consent statement

Written informed consent for participation was obtained from all participants in the ADNI cohort.

## Availability of data and materials

Data used in preparation for this study were obtained from the ADNI database (adni.loni.usc.edu) via data sharing agreements. The participant-level original and preprocessed data cannot be made publicly accessible due to restrictions set by the ADNI. All data supporting the findings described in this paper are available within the paper, in the Supplementary Information and from the corresponding author upon reasonable request.

## Use of AI statement

The Deepseek-R1 AI system wasemployed for linguistic refinement during manuscript preparation, specifically enhancing grammatical precision and stylistic coherence, while remaining uninvolved in data generation processes or preliminary manuscript drafting.

## Funding

This study received funding support from the 10.13039/501100001809National Natural Science Foundation of China (No. 82371435 to W.Y.R.) and Chongqing Municipal Health and Healthy Commission (Chongqing High-Level Medical Talent Program for Young and Middle-Aged Scholars No. YXGD202506 to W.Y.R.).

## CRediT authorship contribution statement

**Zheng-Bin Liao:** Writing – review & editing, Writing – original draft, Investigation. **Zi-Cheng Hu:** Formal analysis, Data curation. **Gui-Hua Zeng:** Formal analysis. **Jia Chen:** Visualization. **Xin-Peng Li:** Visualization. **Yu-Hui Liu:** Writing – review & editing. **Xiu-Qing Yao:** Writing – original draft, Conceptualization. **Ye-Ran Wang:** Writing – original draft, Conceptualization.

## Declaration of competing interest

The authors declare the following financial interests/personal relationships which may be considered as potential competing interests: Ye-Ran Wang reports financial support was provided by National Natural Science Foundation of China. If there are other authors, they declare that they have no known competing financial interests or personal relationships that could have appeared to influence the work reported in this paper.

## Appendix Supplementary materials

Image, application 1
