# Supplementary material for: The association between omega-3 supplementation and cognitive decline in older adults
Source: J Prev Alzheimers Dis. 2026 Apr 17;13(6):100569. doi: 10.1016/j.tjpad.2026.100569 (PMC13099475; doi:10.1016/j.tjpad.2026.100569)
Supplement: Supplementary file 1 [file mmc1.pdf]

Supplementary Materials for

The Association Between Omega-3 Supplementation and Cognitive Decline in Older Adults

Zheng-Bin Liao *et al.*

*Corresponding author. Email:wangyeran@hospital.cqmu.edu.cn

This PDF file includes:

Figures S1 to S3.

Tables S1 to S4.

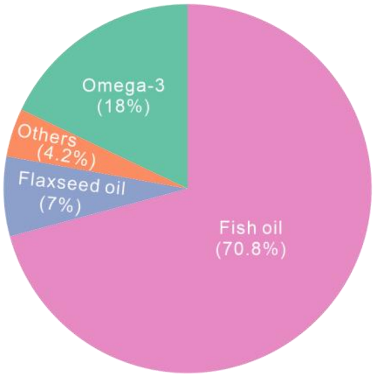

Figure S1. Types of omega-3 in this cohort. Distribution of self-reported supplement types. Data are based on self-reported supplement use recorded at ADNI follow-up visits.

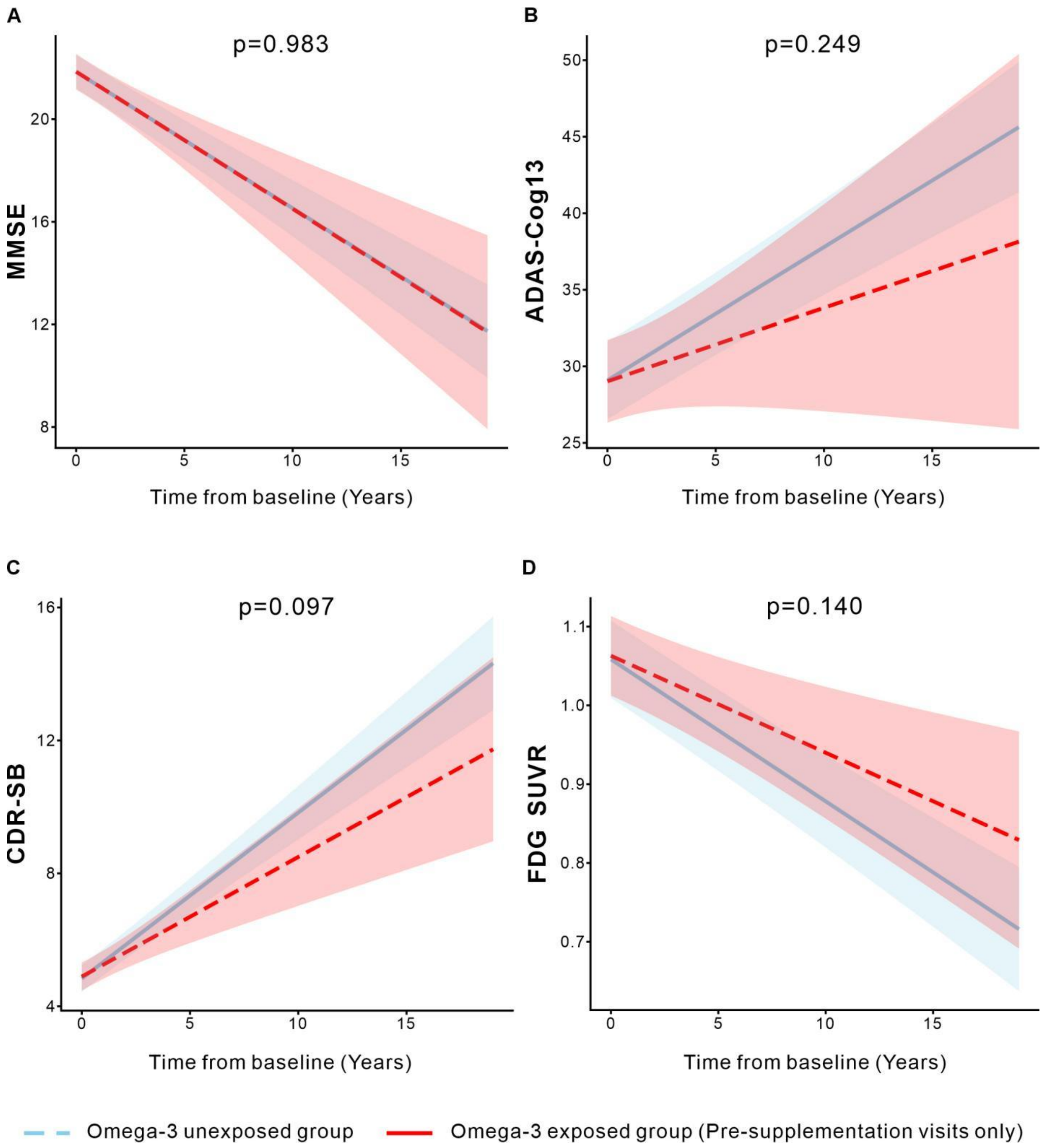

Figure S2. Longitudinal pre-supplementation trajectories of cognitive scores and FDG metabolism in future omega-3 users showed no differences with those of matched non-users. Trajectories for future omega-3 users (red) and matched non-users (blue) are shown for MMSE (A), ADAS-Cog13 (B), CDR-SB (C), and Meta-ROI FDG SUVR (D), with lines representing linear mixed-effects model fits and shaded areas indicating 95% confidence intervals. All analyses were adjusted for age, sex, APOE ε4 status, and diagnosis. MMSE, Mini-

Mental State Examination; ADAS-Cog13, Alzheimer's Disease Assessment Scale-Cognitive Subscale 13; CDR-SB, Clinical Dementia Rating-Sum of Boxes; FDG, fluorodeoxyglucose; SUVR, standardized uptake value ratio; Meta-ROI, meta-region of interest.

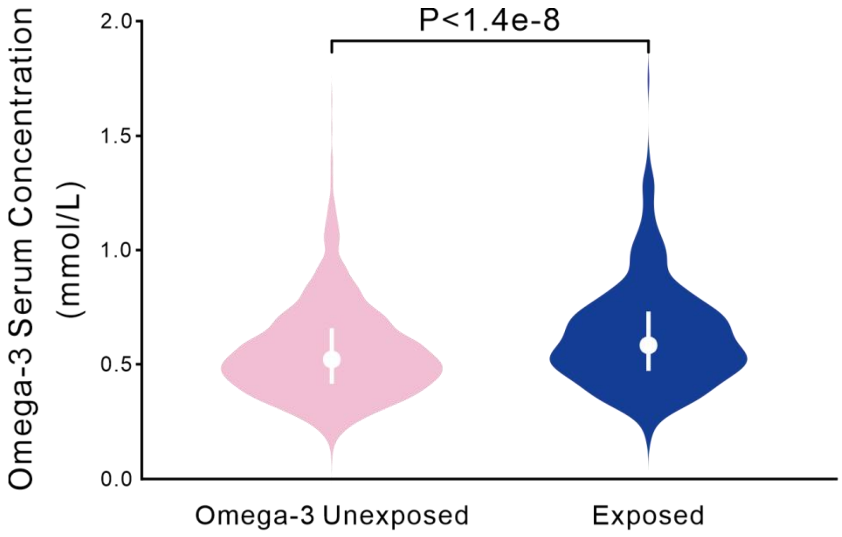

Figure S3. Omega-3 serum concentration is significantly elevated in the exposed group compared to the unexposed group. Violin plots depict the distribution of omega-3 serum levels in each group. The white vertical bars indicate interquatile range (IQR), and the white dots represent the median. The p value was derived from linear mixed effects model. The model structure is as follows: $model: omega - 3\ concentrations: Omega - 3\ exposure\ status + Age + Sex + APOE\ \varepsilon 4\ status + (1 \mid Individual)$.

Table S1. Baseline characteristics of participants before propensity score matching.

	Group			
Characteristic	Omega-3 exposed N = 1,541	Non-exposed N = 273	SMD	P.value
Age,median(IQR,range)	73.8 (68.8, 78.9)	73.0 (69.3, 77.4)	0.09	0.222
Gender,n(%)			0.13	0.065
Male	819 (53.1%)	162 (59.3%)		
Female	722 (46.9%)	111 (40.7%)		
APOE ε4,n(%)			0.08	0.264
No	811 (52.6%)	154 (56.4%)		
Yes	730 (47.4%)	119 (43.6%)		
Diagnosis,n(%)			0.59	0.002
CN	446 (28.9%)	79 (28.9%)		
SMC	79 (5.1%)	3 (1.1%)		
EMCI	206 (13.4%)	65 (23.8%)		
MCI	363 (23.6%)	95 (34.8%)		
LMCI	137 (8.9%)	14 (5.1%)		
AD	310 (20.1%)	17 (6.2%)		
Education,median(IQR,range)	16.0 (14.0, 18.0)	16.0 (14.0, 18.0)	-0.01	0.814
Missing	1,157	104		
ADAS-Cog13,median(IQR,range)	13.0 (8.0, 20.0)	12.0 (8.0, 17.0)	0.23	0.169
Missing	626	167		
FDG SUVR,median(IQR,range)	1.2 (1.1, 1.3)	1.2 (1.1, 1.3)	-0.22	0.010
Missing	422	82		
Stroke,n(%)			0.08	>0.999
No	846 (99.6%)	56 (100.0%)		
Yes	3 (0.4%)	0 (0.0%)		
Missing	692	217		
Parkinson,n(%)			0.03	0.539
No	913 (99.3%)	106 (99.1%)		

	Group			
Characteristic	Omega-3 exposed<br>N = 1,541	Non-exposed<br>N = 273	SMD	P.value
Yes	6 (0.7%)	1 (0.9%)		
Missing	622	166		

Note. Data are presented as median (interquartile range) for continuous variables and counts (%) for categorical variables. Group comparisons were conducted using the Mann–Whitney U test or Fisher's exact test, as appropriate. Standardized mean differences (SMD) were also calculated to assess covariate balance after matching, with SMD < 0.1 indicating adequate balance.

Abbreviations: IQR, interquartile range; *APOE*, apolipoprotein E; CN, cognitively normal; EMCI, early mild cognitive impairment; LMCI, late mild cognitive impairment; SMC, significant memory concern; AD, Alzheimer's disease; ADAS-Cog13, Alzheimer's Disease Assessment Scale-Cognitive subscale 13; FDG, fluorodeoxyglucose; SUVR, standardized uptake value ratio.

Table S2. The relationship of omega-3 supplementation with 68 ROI Aβ deposition.

Brain region	Hemisphere	P-value	FDR p-value
CAUDALANTERIORCINGULATE	Left	0.280	0.937
CAUDALANTERIORCINGULATE	Right	0.507	0.937
CAUDALMIDDLEFRONTAL	Left	0.860	0.937
CAUDALMIDDLEFRONTAL	Right	0.699	0.937
FRONTALPOLE	Left	0.814	0.937
FRONTALPOLE	Right	0.958	0.972
ISTHMUSCINGULATE	Left	0.365	0.937
ISTHMUSCINGULATE	Right	0.295	0.937
LATERALORBITOFRONTAL	Left	0.643	0.937
LATERALORBITOFRONTAL	Right	0.800	0.937
MEDIALORBITOFRONTAL	Left	0.424	0.937
MEDIALORBITOFRONTAL	Right	0.466	0.937
PARSORBITALIS	Left	0.803	0.937
PARSORBITALIS	Right	0.832	0.937
POSTERIORCINGULATE	Left	0.718	0.937
POSTERIORCINGULATE	Right	0.313	0.937
ROSTRALANTERIORCINGULATE	Left	0.247	0.937
ROSTRALANTERIORCINGULATE	Right	0.246	0.937
ROSTRALMIDDLEFRONTAL	Left	0.796	0.937
ROSTRALMIDDLEFRONTAL	Right	0.508	0.937
SUPERIORFRONTAL	Left	0.634	0.937
SUPERIORFRONTAL	Right	0.717	0.937
INSULA	Left	0.894	0.937
INSULA	Right	0.523	0.937
CUNEUS	Left	0.761	0.937
CUNEUS	Right	0.436	0.937

Brain region	Hemisphere	P-value	FDR p-value
LATERALOCCIPITAL	Left	0.848	0.937
LATERALOCCIPITAL	Right	0.933	0.962
LINGUAL	Left	0.841	0.937
LINGUAL	Right	0.888	0.937
PERICALCARINE	Left	0.623	0.937
PERICALCARINE	Right	0.737	0.937
BANKSSTS	Left	0.513	0.937
BANKSSTS	Right	0.440	0.937
PARACENTRAL	Left	0.571	0.937
PARACENTRAL	Right	0.781	0.937
PARSOPERCULARIS	Left	0.768	0.937
PARSOPERCULARIS	Right	0.421	0.937
PARSTRIANGULARIS	Left	0.582	0.937
PARSTRIANGULARIS	Right	0.449	0.937
POSTCENTRAL	Left	0.591	0.937
POSTCENTRAL	Right	0.973	0.973
PRECENTRAL	Left	0.614	0.937
PRECENTRAL	Right	0.846	0.937
INFERIORPARIETAL	Left	0.541	0.937
INFERIORPARIETAL	Right	0.701	0.937
PRECUNEUS	Left	0.563	0.937
PRECUNEUS	Right	0.712	0.937
SUPERIORPARIETAL	Left	0.808	0.937
SUPERIORPARIETAL	Right	0.700	0.937
SUPRAMARGINAL	Left	0.896	0.937
SUPRAMARGINAL	Right	0.717	0.937
ENTORHINAL	Left	0.326	0.937

Brain region	Hemisphere	P-value	FDR p-value
ENTORHINAL	Right	0.627	0.937
FUSIFORM	Left	0.399	0.937
FUSIFORM	Right	0.405	0.937
INFERIORTEMPORAL	Left	0.499	0.937
INFERIORTEMPORAL	Right	0.515	0.937
MIDDLETEMPORAL	Left	0.842	0.937
MIDDLETEMPORAL	Right	0.850	0.937
PARAHIPPOCAMPAL	Left	0.272	0.937
PARAHIPPOCAMPAL	Right	0.064	0.937
SUPERIORTEMPORAL	Left	0.782	0.937
SUPERIORTEMPORAL	Right	0.620	0.937
TEMPORALPOLE	Left	0.126	0.937
TEMPORALPOLE	Right	0.198	0.937
TRANSVERSETEMPORAL	Left	0.819	0.937
TRANSVERSETEMPORAL	Right	0.728	0.937

Note. Linear mixed-effects models were employed to assess the association between omega-3 supplementation and the longitudinal change in regional Aβ deposition (as measured by FBP SUVR). The models included fixed effects for time, omega-3 supplementation, their interaction, and were adjusted for age, sex, *APOE* ε4 status, and diagnosis. P-values are derived from the interaction term between time and omega-3 supplementation, which tests whether the rate of Aβ accumulation differs between groups. FDR correction was applied using the Benjamini-Hochberg (BH) procedure, with a significance threshold set at 0.05.

Abbreviations: *ROI, region of interest; APOE*, apolipoprotein E; FBP, ^{18}F-florbetapir; SUVR, standardized uptake value ratio; Aβ, amyloid-β; FDR, false discovery rate.

Table S3. The relationship of omega-3 supplementation with 68 ROI tau aggregation.

Brain region	Hemisphere	P-value	FDR p-value
CAUDALANTERIORCINGULATE	Left	0.396	0.945
CAUDALANTERIORCINGULATE	Right	0.858	0.945
CAUDALMIDDLEFRONTAL	Left	0.630	0.945
CAUDALMIDDLEFRONTAL	Right	0.549	0.945
FRONTALPOLE	Left	0.589	0.945
FRONTALPOLE	Right	0.975	0.998
ISTHMUSCINGULATE	Left	0.575	0.945
ISTHMUSCINGULATE	Right	0.160	0.945
LATERALORBITOFRONTAL	Left	0.831	0.945
LATERALORBITOFRONTAL	Right	0.873	0.945
MEDIALORBITOFRONTAL	Left	0.628	0.945
MEDIALORBITOFRONTAL	Right	0.461	0.945
PARSOPERCULARIS	Left	0.826	0.945
PARSOPERCULARIS	Right	0.778	0.945
PARSORBITALIS	Left	0.635	0.945
PARSORBITALIS	Right	0.657	0.945
PARSTRIANGULARIS	Left	0.504	0.945
PARSTRIANGULARIS	Right	0.920	0.962
POSTERIORCINGULATE	Left	0.344	0.945
POSTERIORCINGULATE	Right	0.729	0.945
ROSTRALANTERIORCINGULATE	Left	0.890	0.946
ROSTRALANTERIORCINGULATE	Right	0.758	0.945
ROSTRALMIDDLEFRONTAL	Left	0.738	0.945
ROSTRALMIDDLEFRONTAL	Right	0.413	0.945
SUPERIORFRONTAL	Left	0.421	0.945

Brain region	Hemisphere	P-value	FDR p-value
SUPERIORFRONTAL	Right	0.340	0.945
INSULA	Left	0.485	0.945
INSULA	Right	0.876	0.945
CUNEUS	Left	0.482	0.945
CUNEUS	Right	0.467	0.945
LATERALOCCIPITAL	Left	0.051	0.945
LATERALOCCIPITAL	Right	0.356	0.945
LINGUAL	Left	0.295	0.945
LINGUAL	Right	0.498	0.945
PERICALCARINE	Left	0.192	0.945
PERICALCARINE	Right	0.350	0.945
INFERIORPARIETAL	Left	0.449	0.945
INFERIORPARIETAL	Right	0.748	0.945
PARACENTRAL	Left	0.096	0.945
PARACENTRAL	Right	0.020	0.696
POSTCENTRAL	Left	0.168	0.945
POSTCENTRAL	Right	0.372	0.945
PRECENTRAL	Left	0.339	0.945
PRECENTRAL	Right	0.232	0.945
PRECUNEUS	Left	0.143	0.945
PRECUNEUS	Right	0.503	0.945
SUPERIORPARIETAL	Left	0.372	0.945
SUPERIORPARIETAL	Right	0.788	0.945
SUPRAMARGINAL	Left	0.725	0.945
SUPRAMARGINAL	Right	0.517	0.945
BANKSSTS	Left	0.296	0.945

Brain region	Hemisphere	P-value	FDR p-value
BANKSSTS	Right	0.805	0.945
ENTORHINAL	Left	0.018	0.696
ENTORHINAL	Right	0.283	0.945
FUSIFORM	Left	0.383	0.945
FUSIFORM	Right	0.998	0.998
INFERIORTEMPORAL	Left	0.812	0.945
INFERIORTEMPORAL	Right	0.532	0.945
MIDDLETEMPORAL	Left	0.992	0.998
MIDDLETEMPORAL	Right	0.438	0.945
PARAHIPPOCAMPAL	Left	0.218	0.945
PARAHIPPOCAMPAL	Right	0.514	0.945
SUPERIORTEMPORAL	Left	0.873	0.945
SUPERIORTEMPORAL	Right	0.576	0.945
TEMPORALPOLE	Left	0.442	0.945
TEMPORALPOLE	Right	0.525	0.945
TRANSVERSETEMPORAL	Left	0.750	0.945
TRANSVERSETEMPORAL	Right	0.665	0.945

Note. Linear mixed-effects models were employed to assess the association between omega-3 supplementation and the longitudinal change in regional tau aggregation (as measured by FTP SUVR). The models included fixed effects for time, omega-3 supplementation, their interaction, and were adjusted for age, sex, *APOE* ε4 status, and diagnosis. P-values are derived from the interaction term between time and omega-3 supplementation, which tests whether the rate of tau aggregation differs between groups. FDR correction was applied using the Benjamini-Hochberg (BH) procedure, with a significance threshold set at 0.05.

Abbreviations: FTP, ^{18}F-flortaucipir; SUVR, standardized uptake value ratio; *APOE*, apolipoprotein E; FDR, false discovery rate.

Table S4. The relationship of omega-3 supplementation with 68 ROI GMV.

Brain region	Hemisphere	P-value	FDR p-value
PARACENTRAL	Left	0.416	0.681
PARACENTRAL	Right	0.847	0.926
POSTCENTRAL	Left	0.496	0.681
POSTCENTRAL	Right	0.841	0.926
PRECENTRAL	Left	0.907	0.930
PRECENTRAL	Right	0.392	0.681
CAUDALANTERIORCINGULATE	Left	0.073	0.424
CAUDALANTERIORCINGULATE	Right	0.315	0.637
CAUDALMIDDLEFRONTAL	Left	0.084	0.424
CAUDALMIDDLEFRONTAL	Right	0.475	0.681
FRONTALPOLE	Left	0.136	0.498
FRONTALPOLE	Right	0.283	0.626
ISTHMUSCINGULATE	Left	0.215	0.562
ISTHMUSCINGULATE	Right	0.006	0.212
PARSORBITALIS	Left	0.586	0.767
PARSORBITALIS	Right	0.500	0.681
POSTERIORCINGULATE	Left	0.073	0.424
POSTERIORCINGULATE	Right	0.009	0.215
ROSTRALANTERIORCINGULATE	Left	0.632	0.782
ROSTRALANTERIORCINGULATE	Right	0.285	0.626
ROSTRALMIDDLEFRONTAL	Left	0.455	0.681
ROSTRALMIDDLEFRONTAL	Right	0.930	0.930
SUPERIORFRONTAL	Left	0.765	0.882
SUPERIORFRONTAL	Right	0.762	0.882
INSULA	Left	0.546	0.728
INSULA	Right	0.816	0.925

Brain region	Hemisphere	P-value	FDR p-value
CUNEUS	Left	0.466	0.681
CUNEUS	Right	0.437	0.681
LATERALOCCIPITAL	Left	0.134	0.498
LATERALOCCIPITAL	Right	0.006	0.212
LINGUAL	Left	0.144	0.498
LINGUAL	Right	0.025	0.286
LATERALORBITOFRONTAL	Left	0.480	0.681
LATERALORBITOFRONTAL	Right	0.311	0.637
MEDIALORBITOFRONTAL	Left	0.489	0.681
MEDIALORBITOFRONTAL	Right	0.331	0.637
PARSOPERCULARIS	Left	0.920	0.930
PARSOPERCULARIS	Right	0.252	0.592
PARSTRIANGULARIS	Left	0.598	0.767
PARSTRIANGULARIS	Right	0.898	0.930
PERICALCARINE	Left	0.493	0.681
PERICALCARINE	Right	0.738	0.882
INFERIORPARIETAL	Left	0.628	0.782
INFERIORPARIETAL	Right	0.147	0.498
PRECUNEUS	Left	0.756	0.882
PRECUNEUS	Right	0.214	0.562
SUPERIORPARIETAL	Left	0.087	0.424
SUPERIORPARIETAL	Right	0.468	0.681
SUPRAMARGINAL	Left	0.917	0.930
SUPRAMARGINAL	Right	0.364	0.669
BANKSSTS	Left	0.155	0.502
BANKSSTS	Right	0.474	0.681
ENTORHINAL	Left	0.197	0.558

Brain region	Hemisphere	P-value	FDR p-value
ENTORHINAL	Right	0.125	0.498
FUSIFORM	Left	0.104	0.471
FUSIFORM	Right	0.064	0.424
INFERIORTEMPORAL	Left	0.238	0.577
INFERIORTEMPORAL	Right	0.025	0.286
MIDDLETEMPORAL	Left	0.082	0.424
MIDDLETEMPORAL	Right	0.078	0.424
PARAHIPPOCAMPAL	Left	0.018	0.286
PARAHIPPOCAMPAL	Right	0.337	0.637
SUPERIORTEMPORAL	Left	0.321	0.637
SUPERIORTEMPORAL	Right	0.189	0.558
TEMPORALPOLE	Left	0.053	0.424
TEMPORALPOLE	Right	0.225	0.566
TRANSVERSETEMPORAL	Left	0.164	0.507
TRANSVERSETEMPORAL	Right	0.858	0.926

Note. Linear mixed-effects models were employed to assess the association between omega-3 supplementation and the longitudinal change in regional GMV. The models included fixed effects for time, omega-3 supplementation, their interaction, and were adjusted for age, sex, *APOE* ε4 status, and diagnosis. P-values are derived from the interaction term between time and omega-3 supplementation, which tests whether the rate of GMV atrophy differs between groups. FDR correction was applied using the Benjamini-Hochberg (BH) procedure, with a significance threshold set at 0.05.

Abbreviations: *APOE*, apolipoprotein E; FDR, false discovery rate; GMV, gray matter volume.
